# Indications that "codon boundaries" are physico-chemically defined and that protein-folding information is contained in the redundant exon bases

**DOI:** 10.1186/1742-4682-3-28

**Published:** 2006-08-07

**Authors:** Jan Charles Biro

**Affiliations:** 1 Homulus Foundation, San Francisco, CA 94 105, USA

## Abstract

**Background:**

All the information necessary for protein folding is supposed to be present in the amino acid sequence. It is still not possible to provide specific *ab initio *structure predictions by bioinformatical methods. It is suspected that additional folding information is present in protein coding nucleic acid sequences, but this is not represented by the known genetic code.

**Results:**

Nucleic acid subsequences comprising the 1st and/or 3rd codon residues in mRNAs express significantly higher free folding energy (FFE) than the subsequence containing only the 2nd residues (*p *< 0.0001, *n *= 81). This periodic FFE difference is not present in introns. It is therefore a specific physico-chemical characteristic of coding sequences and might contribute to unambiguous definition of codon boundaries during translation. The FFEs of the 1st and 3rd residues are additive, which suggests that these residues contain a significant number of complementary bases and that may contribute to selection for local RNA secondary structures in coding regions. This periodic, codon-related structure-formation of mRNAs indicates a connection between the structures of exons and the corresponding (translated) proteins. The folding energy dot plots of RNAs and the residue contact maps of the coded proteins are indeed similar. Residue contact statistics using 81 different protein structures confirmed that amino acids that are coded by partially reverse and complementary codons (Watson-Crick (WC) base pairs at the 1st and 3rd codon positions and translated in reverse orientation) are preferentially co-located in protein structures.

**Conclusion:**

Exons are distinguished from introns, and codon boundaries are physico-chemically defined, by periodically distributed FFE differences between codon positions. There is a selection for local RNA secondary structures in coding regions and this nucleic acid structure resembles the folding profiles of the coded proteins. The preferentially (specifically) interacting amino acids are coded by partially complementary codons, which strongly supports the connection between mRNA and the corresponding protein structures and indicates that there is protein folding information in nucleic acids that is not present in the genetic code. This might suggest an additional explanation of codon redundancy.

## Background

The protein folding problem has been one of the grand challenges in computational molecular biology. The problem is to predict the native three-dimensional structure of a protein from its amino acid sequence. It is widely believed that the amino acid sequence contains all the information necessary to make up the correct three-dimensional structure, since protein folding is apparently thermodynamically determined; i.e., given a proper environment, a protein will fold up spontaneously. This is called Anfinsen's thermodynamic principle [1].

The thermodynamic principle has been confirmed many times on many different kinds of proteins in vitro. Critics says that the in vivo chemical conditions are different from those in vitro, the correct folding is determined by interactions with other molecules (chaperons, hormones, substrate, etc.) and protein folding is much more complex than re-naturation of denatured poly-amino acids. The fact that many naturally-occurring proteins fold reliably and quickly to their native state, despite the astronomical number of possible configurations, has come to be known as Levinthal's Paradox [2].

Anfinsen's principle was formulated in the 1960s using purely chemical experiments and a lot of intuition. Today, many sequences and structures are available to establish a logical and understandable link between sequence, structure and function. But it is still not possible to predict the structure (or a range of possible structures) correctly from the sequence alone, ab initio and in silico [3].

There are two potential, external sources of additional and specific protein folding information: (a) the chaperons (other proteins that assist in the folding of proteins and nucleic acids [4]; and (b) the protein-coding nucleic acid sequences themselves (which are templates for protein synthesis, but are not defined as chaperons).

The idea that the nucleotide sequence itself could modulate translation and hence affect co-translational folding and assembly of proteins has been investigated in a number of studies [5-7]. Studies on the relationships between synonymous codon usage and protein secondary structural units are especially popular [8-10]. The genetic code is redundant (61 codons code 20 amino acids) and as many as 6 synonymous codons can code the same amino acid (Arg, Leu, Ser). The "wobble" base has no effect on the meaning of most codons, but nevertheless codon usage (wobble usage) is not randomly defined [11,12] and there are well-known, stable species-specific differences in codon usage. It seems logical to search for some meaning (biological purpose) for the wobble bases and try to associate them with protein folding.

Another observation concerning the code redundancy dilemma is that there is a widespread selection (preference) for local RNA secondary structure in protein coding regions [13]. A given protein can be encoded by a large number of distinct mRNA species, potentially allowing mRNAs to optimize desirable RNA structural features simultaneously, in addition to their protein coding function. The immediate question is whether there is some logical connection between the possible optimal RNA structures and the possible optimal biologically active protein structures.

## Methods

Single-stranded RNA molecules can form local secondary structures through the interactions of complementary segments. Watson-Crick (WC) base pair formation lowers the average free energy, d*G*, of the RNA and the magnitude of change is proportional to the number of base pair formations. Therefore the free folding energy (FFE) is used to characterize the local complementarity of nucleic acids [13]. The free folding energy is defined as FFE = (d*G*_shuffled _- d*G*_native_)/*L *× 100, where *L *is the length of the nucleic acid, i.e., free energy difference between native and shuffled (randomized) nucleic acids per 100 nucleotides. Higher positive values indicate stronger bias toward secondary structure in the native mRNA, and negative values indicate bias against secondary structure in the native mRNA.

We used a nucleic acid secondary structure predicting tool, the *mfold *[14] to obtain d*G *values and the lowest d*G *was used to calculate the FFE. The mfold also provided the folding energy dot plots, which are very useful for visualizing the energetically most favored structures in a 2D matrix.

A series of JAVA tools were used: SeqX to visualize the protein structures in 2D as amino acid residue contact maps [15]; SeqForm to select sequence residues in predefined phases (every third in our case) [16]. Structural data were downloaded from PDB [17], NDB [18], and the Integrated Sequence-Structure Database (ISSD) [19].

Structures were generally randomly selected regarding species and biological function (a few exceptions are mentioned in the Results). Care was taken to avoid very similar structures in the selections. A propensity for alpha helices was monitored during selection and structures with very high and very low alpha helix contents were also selected to ensure a wide range of structural representations.

Linear regression analyses and Student's *t*-tests were used for statistical analysis of the results.

## Results

**Figure 1:**
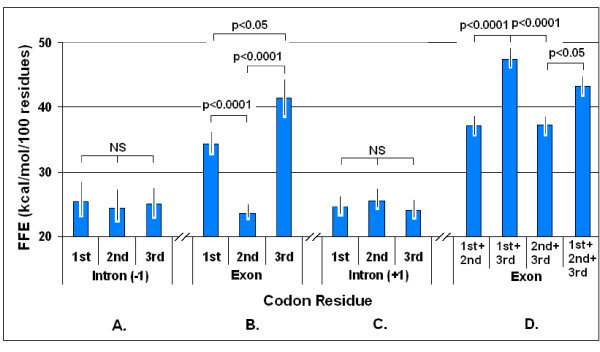
**Free folding energies in different codon residues**. Free folding energies (FFE) were determined in phase-selected subsequences of 81 different genes. The original nucleic acids contained the intact three-letter codons (1^st^+2^nd^+3^rd^). Subsequences were constructed by periodic removal of one letter from the codon and maintaining the other two (1^st^+2^nd^, 1^st^+3^rd^, 2^nd^+3^rd^) or removing two letters and maintaining only one (1^st^, 2^nd^, 3^rd^). Distinction was made between exons (B and D) and the preceding (-1, A) and following (+1, C) sequences (introns). The d*G *values were determined by *mfold *and the FFE was calculated. Each bar represents the mean ± SEM, *n *= 81.

A selection of 81 different protein structures together with the corresponding protein and coding sequences was used for this study. These 81 proteins represented different (randomly selected) species and different (also randomly selected) protein functions and therefore the results might be regarded as more generally valid. The propensity for different secondary structure elements was recorded (as annotated in different databases). The proportion of alpha helices ranged from 0 to 90% in the 81 proteins and showed a significant negative correlation to the proportion of beta sheets (not shown). The coding sequences were phase separated by SeqForm into three subsequences, each containing only the 1^st^, 2^nd ^and 3^rd ^letters of the codons. Similar phase separation was made for intronic sequences immediately before and after the exon. There are, of course, no known codons in the intronic sequences, therefore we continued the same phase that we applied to the exon, assuming that this kind of selection is correct, and maintained the denotation of the phase even for non-coding regions. Subsequences corresponding to the 1^st ^and 3^rd ^codon letters in the coding regions had significantly higher FFEs than subsequences corresponding to the 2^nd ^codon letters. No such difference was seen in non-coding regions (Figure 1A–C).

Higher FFEs in subsequences of 1^st ^and 3^rd ^codon residues than in the 2^nd ^indicate the presence of a larger number of complementary bases at the right positions of these subsequences. However, this might be the case only because the first and last codon residues form simpler subsequences and contain longer repeats of the same nucleotide than the 2^nd ^residues. This would not be surprising for the 3^rd ^(wobble) base but would not be expected for the 1^st ^residue, even though it is known that the central codon letters are the most important for distinguishing among amino acids (as shown in the in the *Common Periodic Table of Codons and Amino Acids *[20]). It is more significant that the FFEs for the 1^st ^and 3^rd ^residues are additive and together they represent the entire FFE of the intact mRNA (Figure 1D).

**Figure 2:**
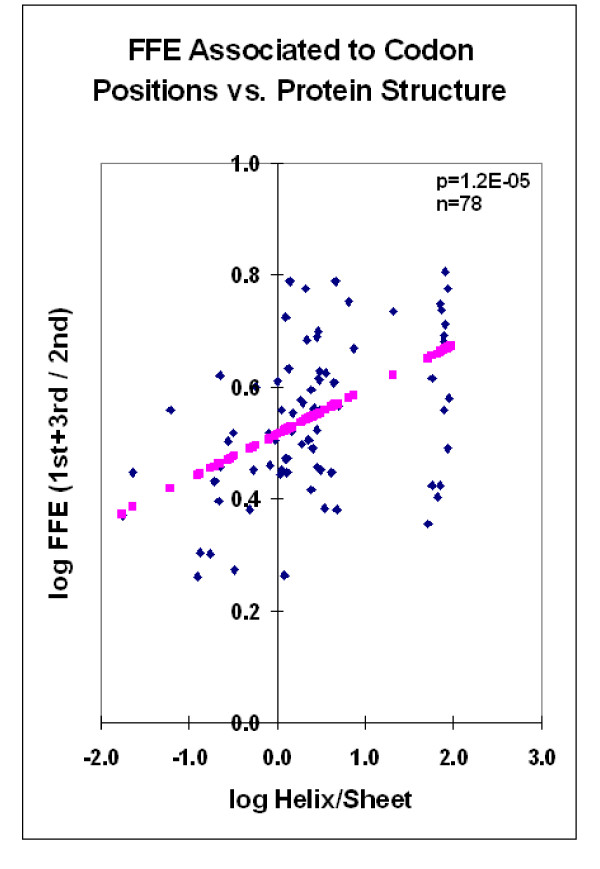
**FFE associated with codon positions vs. protein structure**. Free Folding Energies associated with 1^st^, 2^nd ^and 3^rd ^codon residues in 78 different mRNA sequences were calculated and compared to the helix/sheet ratios of the corresponding protein structures. Linear regression analyses, where pink symbols represent the linear regression line.

There is a correlation between the protein structure and the FFEs associated with codon residues. This correlation is especially prominent when the FFE ratios are compared to the helix/sheet ratios (Figure 2).

**Figure 3:**
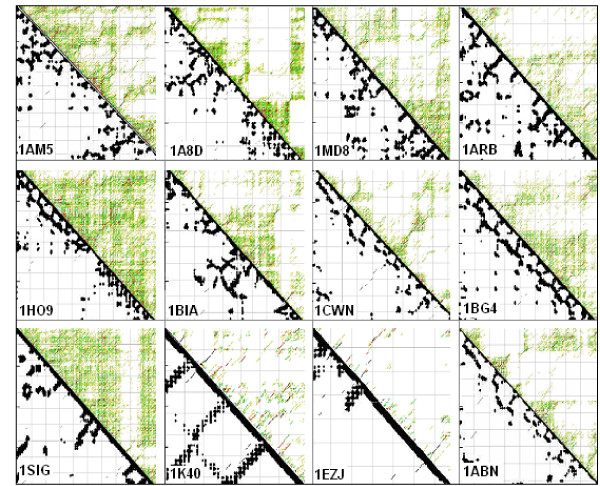
**Comparison of protein and corresponding mRNA structures**. Residue contact maps (RCM) were obtained from the PBD files of protein structures using the SeqX tool (left triangles). Energy dot plots (EDP) for the coding sequences were obtained using the mfold tool (right triangles). The two kinds of maps were aligned along a common left diagonal axis to make possible an easy visual comparison of the different kind of representations. The black dots in the RCMs indicate amino acids that are within 6Å of each other in the protein structure. The colored (grass-like) areas in the EDPs indicate the energetically mostly likely RNA interactions (color code in increasing order: yellow, green, red, black).

The unique, codon-related FFE pattern and its correlation to alpha helix content suggested some similarity between protein structures and the possible structures of the coding sequences. This possibility was examined by visual comparison of 16 randomly selected protein residue contact maps and the energy dot plots of the corresponding RNAs. We could see similarities between the two different kinds of maps (Figure 3). However, this type of comparison is not quantitative and direct statistical evaluation is not possible.

**Figure 4:**
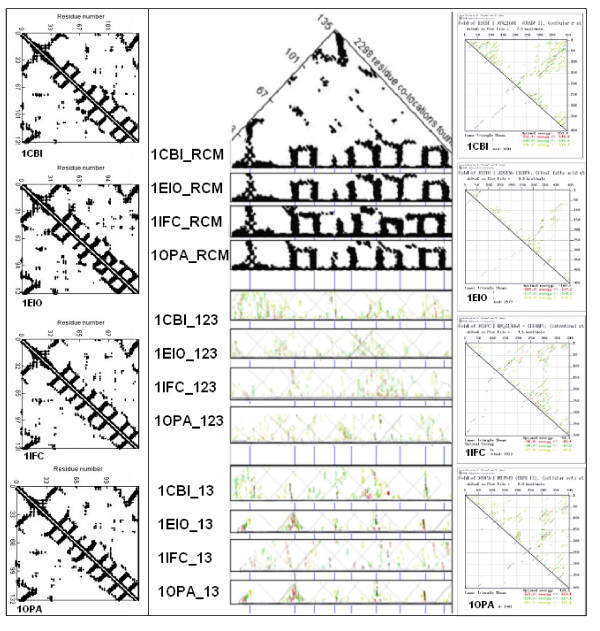
**Comparison of protein and mRNA secondary structures**. Residue contact maps (RCM) were obtained from the PBD files of 4 protein structures (1CBI, 1EIO, 1IFC, 1OPA) using the SeqX tool (left column). Energy dot plots (EDP) for the coding sequences were obtained using the mfold tool (right column). The left diagonal portions of these two kinds of maps are compared in the central part of the figure. Blue horizontal lines in the background correspond to the main amino acid co-location sites in the RCM. Intact RNA (123) as well as subsequences containing only the 1st and 3rd codon letters (13) are compared. The black dots in the RCMs indicate amino acids that are within 6Å of each other in the protein structure. The colored (grass-like) areas in the EDPs indicate the energetically most likely RNA interactions (color code in increasing order: yellow, green, red, black).

Another similar, but still not quantitative, comparison of protein and coding structures was performed on four proteins that are known to have very similar 3D structures although their primary structures (sequences), and their mRNA sequences, are less than 30% similar. These four proteins exemplify the fact that the tertiary structures of proteins are much more conserved than the amino acid sequences. We asked whether this is also true for the RNA structures and sequences. We found that there are signs of conservation even in the RNA secondary structure (as indicated by the energy dot plots) and there are similarities between the protein and nucleic acid structures (Figure 4). Comparisons of the protein residue contact map with the nucleic acid folding maps suggest similarities between the 3D structures of these different kinds of molecules. However, this is a semi-quantitative method.

More direct statistical support might be obtained by analyzing and comparing residue co-locations in these structures. Assume that the structural unit of mRNA is a tri-nucleotide (codon) and the structural unit of the protein is the amino acid. The codon may form a secondary structure by interacting with other codons according to the WC base complementary rules, and contribute to the formation of a local double helix. The 5'-A1U2G3-3' sequence (Met, M codon) forms a perfect double string with the 3'-U3A2C1-5' sequence (His, H codon, reverse and complementary reading). Suboptimal complexes are 5'-A1X2G3-3' partially complemented by 3'-U3X2C1-5' (AAG, Lys; AUG, Met; AGG, Arg; ACG, Thr; and CAU, His; CUU, Leu; CGU, Arg; CCU, Pro, respectively).

**Figure 5:**
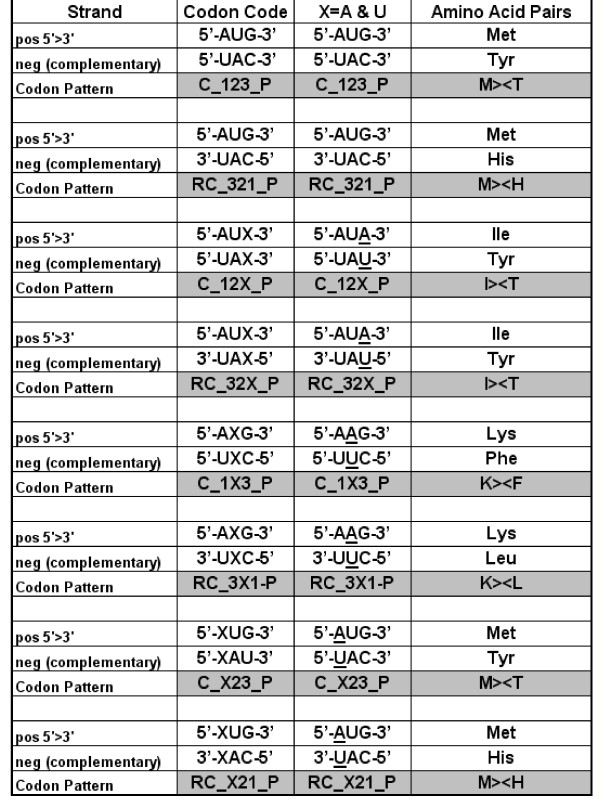
**Amino acid pairs coded by complementary codons**. Two optimal (perfect) and six suboptimal (partial) codon complementarity situations (codon codes) are listed. In the perfect complementarity situation a codon (AUG), which is transcribed from the sense (pos) DNA strand in the 5'>3' direction, is complemented with the UAC codon that is transcribed from the antisense (neg) DNA strand in complementary (C-123) or reverse-complementary (RC-321) orientations. In the suboptimal codon codes, one codon residue is undefined (X) and may or may not complemented in the corresponding codon on the negative strand in that residue position. Translation of the codon and its complementary pair will result in different amino acid pairs, depending on the codon pattern. This is illustrated in examples where the undefined X residues uniformly replaced by A in the positive and U in the negative strands. For example, the meaning of 5'-AAG-3'/3'-UUC-5' (from the D_1X3/RC_3X1 codon code pattern) is that this codon pair will be translated into the amino acids Lys (K) and Leu (L) and will result in K><L residue pairs. Letter -P at the end of a codon pattern indicates the presence (-P), in contrast to the non-presence (-N), of that particular codon code to determine a specific amino acid co-location in a concrete protein structure (this is used in Figure 6).

I searched for some pattern in the codons of co-locating amino acids and analyzed the frequencies of the 8 possible patterns in the 64 nucleic acid triplets (Figure 5). The codons were either complementary to each other in all three (-123-) or at least 2 (-12X-, 1X3-, -X23-) codon positions. In these latter cases the codon complementarity was partial, because complementarity was not required for one codon position (X). The complementary codons were translated in the same (5'>3' & 3'>5', only complementary, C) or reversed and complementary (5'>3' & 5'>3', RC) directions.

These perfectly or partially complementary codon patterns, read in direct or opposite directions, defined 8 different ways in which amino acids can be paired on the basis of their codon complementarities: the 8 possible amino acid – amino acid (or protein-protein) interaction codes.

**Figure 6:**
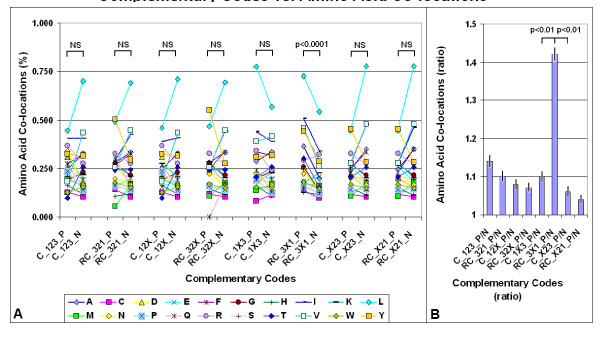
**Complementary codes vs. amino acid co-locations**. A: The propensities for the 400 possible amino acid pairs were monitored in 81 different protein structures with the SeqX tool. The tool detected co-locations when two amino acids were closer than 6Å to each other (neighbors on the same strand were excluded). The total number of co-locations was 34,630. Eight different complementary codes were constructed for the codons (two optimal and six suboptimal). In the two optimal codes all three codon residues (123) were complementary (C) or reverse-complementary (RC) to each other. In the suboptimal codes only two of three codon residues were C or RC to each other (12, 13, 23), while the third was not necessarily complementary (X). (For example, complementary code RC_3X1 means that the first and third codon letters are always complementary (to D_1X3), but not the second, and the possible codons are read in reverse orientation). The 400 co-locations were divided into 20 subgroups corresponding to 20 amino acids (one of the co-locating pairs), each group containing 20 amino acids (corresponding to the other amino acids in each co-locating pair). If the codons of the amino acid pairs followed the predefined complementary code, the co-location was regarded as positive (P); if not, the co-location was regarded as negative (N). Each symbol represents the mean frequency of P or N co-locations corresponding to the indicated amino acid. Paired Student's *t*-test, *n *= 20. B: The ratio of positive (P) and negative (N) co-locations was calculated on data from (A). Each bar represents the mean ± SEM, *n *= 20.

Our experiments with FFE indicate that local nucleic acid structures are formed under this suboptimal condition, i.e., when the 1^st ^and 3^rd ^codon residues are complementary but the 2^nd ^is not. If this is the case, and there is a connection between nucleic acid and protein 3D structures, one might expect that the 4 amino acids coded by 5'-A1X2G3-3' codons will preferentially co-locate with 4 other amino acids coded by 3'-U3X2C1-5' codons. We have constructed 8 different complementary codon combinations and found that the codons of co-locating amino acids are often complementary at the 1^st ^and 3^rd ^positions and follow the D-1X3/RC-3X1 formula but not the 7 other formulae (Figure 6A–B). This means that amino acids that are coded by partially reverse and complementary codons (WC base pairs at the 1^st ^and 3^rd ^codon positions and translated in reverse orientation) are preferentially co-located in protein structures.

## Discussion

It is well known that coding and non-coding DNA sequences (exons/introns) are different and this difference is somehow related to the asymmetry of the codons, i.e. that the third codon letter (wobble) is less important in defining the meaning of the codon than the first and second letters. Many Markov models have been formulated to find this asymmetry and predict coding sequences (genes) de novo. These in silico methods work rather well but not perfectly and some scientists remain unconvinced that codon asymmetry explains the exon-intron differences satisfactorily.

Another codon-related problem is that the well-known, non-overlapping, triplet codon translation process is extremely phase-dependent and there is theoretically no tolerance for any phase shift. There are famous examples of single nucleotide deletions that destroy the meaningful translation of a sequence and are incompatible with life. However, considering the magnitude and complexity of the eukaryotic proteome, the precision of translation is astonishingly good. Such physical precision is not possible without a massive and consistent physico-chemical underpinning. Therefore, discovery of the existence of secondary structure bias (folding energy differences) in coding regions of many organisms [13] was very welcome because it clearly defined codon boundaries on a physico-chemical basis.

Our experiments with free folding energy (FFE) confirmed that this bias exists. In addition, there is a very consistent and very significant pattern of FFE distribution along the nucleotide sequence. Comparing the FFEs of phase-selected subsequences, those subsequences comprising only the 1^st ^or only the 3^rd ^codon letters showed significantly higher FFE than those consisting only of the 2^nd ^letters. This FFE difference was not present in the intronic sequences preceding and following the exons, but it was present in exons from different species. This is an interesting observation because these phenomena might not only distinguish between exons and introns on a physico-chemical basis, but might also clearly define the tri-nucleotide codons and thus the phase of the translation. This codon-related phase-specific variation in FFE may explain why mRNAs have greater negative free folding energies than shuffled or codon choice randomized sequences [21].

Free folding energy in nucleic acids is always associated with WC base pair formation. A higher FFE indicates more WC pairs (presence of complementarity) and a lower FFE indicates fewer WC pairs (less complementarity). The FFEs in the 1^st ^and 3^rd ^codon positions were additive, while the 2^nd ^letter did not contribute to the total FFE; the total FFE of the entire (intact) nucleic acid was the same as that of subsequences containing only the 1^st ^and 3^rd ^codon letters (2^nd ^deleted). This indicates that local RNA secondary structure bias is caused by complementarity of the 1^st ^and 3^rd ^codon residues in local sequences. This partial, local complementarity is more optimal in reverse orientation of the local sequences, as expected with loop formations.

FFEs are obtained by considering free folding energies of substrings that do not represent the real molecule: forcing nucleotides to be consecutive is an extreme methodological approach to measuring the structure features of coding sequences. However, this bioinformatical method has been successfully used by others [13,21]. In addition, the behaviors of the 1^st^, 2^nd ^and 3^rd ^codon bases separately are useful for showing that the 2^nd ^codon position does not have the same significance in the codons as the other two positions [20]. Intronic sequences do not contain codons and consequently thy show no position-related periodic-FFE variation.

It is known that single-stranded RNA molecules can form local secondary structures through the interactions of complementary segments. The novel observation here is that these interactions preferentially involve the 1^st ^and 3^rd ^codon residues. This connection between the RNA secondary structure and codons immediately directs attention toward the question of protein folding and its long-suspected connection to RNA folding [22,23].

Only about one-third (20/64) of the genetic code is used for protein coding, i.e., there is a great excess of information in the mRNA. At the same time, the information carried by amino acids seems to be insufficient (as stated by some scientists) to complete unambiguous protein folding. Therefore, it is believed that the third codon residue (wobble base) contains information additional to that already present in the genetic code. A specialized database, the ISSD [19], was established in an effort to connect different features of protein structure to wobble bases [24] with more or less success.

We found a significant correlation between FFE ratios and the helix/sheet contents of protein structures. It was possible to make direct visual comparison of mRNA structures (as statistically predicted by mfold energy dot-plots) and protein structures (as 2D residue contact maps). This method suggests similarity between nucleic acid and protein structures.

It is known that some complex protein structures are very similar even if there is less than 30% sequence similarity. It was interesting to see whether the same principle might apply to nucleic acids, and structural similarity might exist even when the sequence similarity is low. Furthermore, significant similarity between nucleic acid and protein structures might exist even without translational connection. Structure seems to be more preserved, even in nucleic acids, than sequence. However, although the matrix comparisons are suggestive, they remain semi-quantitative. Better support was necessary.

A working hypotheses grew out of these observations, namely that (a) partial, local reverse-complementarity exists in nucleic acids that form the nucleic acid structure; (b) there is some degree of similarity between the folding of nucleic acids and proteins; (c) protein structure determines the amino acid co-locations; (4) in consequence, amino acids coded by interacting (partially reverse complementary) codons might show preferential co-locations in the protein structures. And it seems to be the case: codons that contain complementary bases at the 1^st ^and 3^rd ^positions and are translated in reverse orientation result in preferentially co-located (interacting) amino acids in the 3D protein structure. Other complementary residue combinations or translation in the same (not reverse) direction (as many as seven combinations in total) did not result in any preferentially co-locating subset of amino acid pairs.

Construction of residue contact maps for protein structures and statistical evaluation of residue co-locations is a frequently used method for visualization and analysis of spatial connections among amino acids [25-27]. The amino acid co-locations in real protein structures are clearly not random [28,29] and therefore residue co-location matrices are often used to assist in the prediction of novel protein structures [30,31]. We have carefully examined the physico-chemical properties of specifically interacting amino acids in and between protein structures, and concluded that these interactions follow the well-known physico-chemical rules of size, charge and hydrophobic compatibility (unpublished data), well in line with Anfinsen's prediction. The recent study supports the conclusion that there is a previously unknown connection between the codons of specifically interacting amino acids; those codons are complementary at the 1^st ^and 3^rd ^(but not the 2^nd^) codon positions.

The idea that sequence complementarity might explain the nature of specific protein-protein interactions is not new and was suggested as long ago as 1981 [32,35,36]. I was never able to confirm my own original theory experimentally, the suggestion of perfect complementarity between codons of interacting amino acids [32,33], though others were more successful [34]. The explanation is that codon complementarity is suboptimal and does not involve the 2^nd ^codon residue. Experimental in vitro confirmation is required to validate this recent theoretical and in silico prediction.

**Availability: **: SeqX, SeqForm.
